# Identifying subtypes of depression in clinician-annotated text: a retrospective cohort study

**DOI:** 10.1038/s41598-021-01954-4

**Published:** 2021-11-17

**Authors:** Benson Kung, Maurice Chiang, Gayan Perera, Megan Pritchard, Robert Stewart

**Affiliations:** 1 Prairie Health, Palo Alto, USA; 2 Institute of Psychiatry, Psychology and Neuroscience, King’s College London, London, UK; 3 South London and Maudsley NHS Foundation Trust, London, UK

**Keywords:** Depression, Diagnosis, Signs and symptoms, Epidemiology

## Abstract

Current criteria for depression are imprecise and do not accurately characterize its distinct clinical presentations. As a result, its diagnosis lacks clinical utility in both treatment and research settings. Data-driven efforts to refine criteria have typically focused on a limited set of symptoms that do not reflect the disorder’s heterogeneity. By contrast, clinicians often write about patients in depth, creating descriptions that may better characterize depression. However, clinical text is not commonly used to this end. Here we show that clinically relevant depressive subtypes can be derived from unstructured electronic health records. Five subtypes were identified amongst 18,314 patients with depression treated at a large mental healthcare provider by using unsupervised machine learning: severe-typical, psychotic, mild-typical, agitated, and anergic-apathetic. Subtypes were used to place patients in groups for validation; groups were found to be associated with future outcomes and characteristics that were consistent with the subtypes. These associations suggest that these categorizations are actionable due to their validity with respect to disease prognosis. Moreover, they were derived with automated techniques that might theoretically be widely implemented, allowing for future analyses in more varied populations and settings. Additional research, especially with respect to treatment response, may prove useful in further evaluation.

## Introduction

Depression affects over 264 million people every year^1^ and is a leading cause of disability worldwide^2^. It is a heterogeneous disorder with a significant diversity of presentations^3^. Common severity scales measure as many as 50 unique symptoms, often with little overlap^4^. To account for this heterogeneity, researchers have worked to refine depression diagnosis by identifying potential subtypes with data-driven approaches.

Prior studies have created subtypes often characterized by severity and combinations of symptoms, such as mild atypical depression or severe depression with anxiety^3–6^. They typically analyze a limited number of depression-related characteristics using common symptom inventories such as the Patient Health Questionnaire-9^7^ or the Quick Inventory of Depressive Symptomatology^8^. However, there is increasing consensus that depression should not be studied in isolation^6,9^. It is often comorbid with other disorders, such as anxiety^10^, and interventions have been shown to be effective across diagnoses^11^. Moreover, biological processes related to mental health are generally associated with multiple disorders^12–14^. These considerations have led to research frameworks that focus on empirically derived biological and behavioral processes, as opposed to consensus-driven criteria, such as the National Institute of Mental Health’s (NIMH) Research Domain Criteria (RDoC) initiative^15^.

Less attention has been placed on subtype identification utilizing electronic health records (EHRs), especially free text fields, where patient presentations are conventionally described in detail. This is in spite of the fact that EHRs can provide data on large sample sizes in real-world settings, where disorders are treated in the context of a patient’s overall mental and physical health. Furthermore, metadata from EHRs has the potential to be automatically derived, analyzed, and fed back into a clinical interface to guide intervention decisions.

Retrospective analyses in other medical fields have shown that data leveraged from EHRs can be valuable. For example, Jensen et al. used free text in EHRs to estimate cancer trajectories, predicting 80% of events in a cohort of 7,741 patients^16^. Madison et al. leveraged multiple data types, including free text from EHRs, to ascertain clinical outcomes, including cohort characteristics, oral therapy usage, treatment progression and response^17^. And Rajkomar, Alvin, et al. combined different data types to build deep learning models that could accurately predict readmission risk, inpatient mortality, and diagnoses^18^. Thus, data recorded from clinicians in unstructured EHRs offer new opportunities to study transdiagnostic constructs that are not limited to a fixed set of features.

This study aimed to create clinically relevant depressive subtypes by leveraging symptom data derived from unstructured fields in EHRs. We used unsupervised machine learning to decompose symptom data derived from clinical text into natural subtypes. A range of service outcomes were then chosen for further analysis of predictive validity. We hypothesized that the subtypes would stratify patients into coherent groups with respect to outcome data.

## Methods

### Participants

Unstructured EHR data were accessed from the South London Maudsley Trust NHS Foundation Trust (SLaM). SLaM provides specialist mental healthcare to approximately 1.3 million residents of four London boroughs, and has used an EHR for all its services since 2006. The Clinical Record Interactive Search (CRIS) data platform was developed between 2007 and 2008 to make de-identified data from SLaM’s EHR available for research within a robust governance framework^19,20^. CRIS data has been substantially enhanced over the last 10 years by a series of natural language processing (NLP) algorithms designed to extract data of interest from free text fields in the EHR^21^. Use of CRIS as a data source for secondary analyses has received IRB approval (Oxford Research Ethics Committee C reference 18/SC/0372); the methods presented here were conducted in compliance with the relevant guidelines. No identifying information was used as a part of this study.

De-identified data from 18,314 patients treated at SLaM from January 1st, 2007 to November 1st, 2018 were analyzed. Patients were included if they received a primary diagnosis of depression (ICD-10 F33 or F32) within the first 3 months of their first face-to-face interaction with SLaM. Demographic information for the total sample is included as a part of Table 1.

**Table 1:** Demographic information for the total sample as well as within patient groups.

	Full sample	Groups 3–5	1	2	3	4	5	*p*-value Full Sample	*p*-value Groups 3–5
**Total Sample**	18,314	12,115	3,140	3,059	4,844	4,291	2,980		
**Gender**								< 0.001	< 0.001
Female	11,377 (62.1)	7825 (64.6)	1849 (58.9)	1703 (55.7)	3441 (71.0)	2500 (58.3)	1884 (63.2)		
Male	6926 (37.8)	4283 (35.4)	1290 (41.1)	1353 (44.2)	1401 (28.9)	1789 (41.7)	1093 (36.7)		
**Race**								< 0.001	0.69
Asian	915 (5.0)	573 (4.7)	151 (4.8)	191 (6.2)	227 (4.7)	218 (5.1)	128 (4.3)		
Black	2728 (14.9)	1709 (14.1)	448 (14.3)	571 (18.7)	670 (13.8)	603 (14.1)	436 (14.6)		
Mixed	400 (2.2)	274 (2.3)	62 (2.0)	64 (2.1)	111 (2.3)	95 (2.2)	68 (2.3)		
Other	1833 (10)	1236 (10.2)	305 (9.7)	292 (9.5)	506 (10.4)	449 (10.5)	281 (9.4)		
White	10,458 (57.1)	6956 (57.4)	1849 (58.9)	1653 (54.0)	2787 (57.5)	2449 (57.1)	1720 (57.7)		
*Ethnicity missing*	1980 (10.8)	1367 (11.3)	325 (10.4)	288 (9.4)	543 (11.2)	477 (11.1)	347 (11.6)		
**Age**								< 0.001	< 0.001
< 18	2352 (12.8)	1750 (14.4)	345 (11.0)	257 (8.4)	772 (15.9)	664 (15.5)	314 (10.5)		
18–34	5951 (32.5)	3954 (32.6)	1032 (32.9)	965 (31.5)	1580 (32.6)	1289 (30.0)	1085 (36.4)		
35–49	4513 (24.6)	2923 (24.1)	833 (26.5)	757 (24.7)	1175 (24.3)	1033 (24.1)	715 (24)		
50–64	2561 (14)	1576 (13)	480 (15.3)	505 (16.5)	620 (12.8)	590 (13.7)	366 (12.3)		
65& +	2934 (16)	1910 (15.8)	449 (14.3)	575 (18.8)	696 (14.4)	714 (16.6)	500 (16.8)		
**Mean deprivation score (SD)**	25.1 (10.2)	25.1 (10.3)	24.8 (10.2)	25.4 (10.1)	25.0 (10.0)	25.2 (10.4)	25.2 (10.2)	0.16	0.05

### Measures

Fifty psychiatric symptoms, which included a range covering psychotic, bipolar and depressive disorders, derived from unstructured EHRs with rules-based algorithms were used to create subtypes. The symptoms are listed in Supplementary eTable 1. The algorithms were developed prior to this study; detailed methodologies and performance metrics for each algorithm are documented by the CRIS NLP service^21^. All algorithms seek to determine whether a patient experienced a symptom or not, excluding irrelevant mentions such as negative statements. A symptom was considered present in a patient if it was extracted from text fields drawn from the first month of clinical contact. These binary variables were used for the subtype generation process described below.

### Outcomes

Predictive validity of the derived subtypes was evaluated with respect to the occurrence of a mental health crisis as a primary outcome. This was defined as any admission to mental health inpatient care or an episode of home-treatment team care, an alternative to the former, within the window between 3 and 15 months after a patient’s first face-to-face encounter with SLaM. In addition, the following secondary outcomes were studied within the same period: (1) occurrence of an emergency room presentation; (2) number of days active to SLaM within the window; (3) number of recorded face-to-face contacts with SLaM clinical staff; (4) mortality within the window excluding deaths after August 6th, 2020; (5) number of years of follow-up.

Additionally, covariates were investigated: age, gender, ethnic group (classified into White, Black, Asian, Mixed, Other), year of first SLaM contact, and neighborhood deprivation (Index of Multiple Deprivation, a standard metric derived from national census data and applied at the level of the Lower Super Output Area, a national administrative unit with an average 1500 residents).

Information from the Health of the Nation Outcome Scales (HoNOS) was also extracted. HoNOS is a clinician-rated instrument composed of 11 scales quantifying different elements of mental health and general function, where each scale is rated between 0 to 4. A score of 2 corresponds to a mild problem; as a result, patients were considered to have a HoNOS-defined problem if they scored between 2 and 4.

Finally, different types of medications received during the window were studied. The results are presented in Supplementary eTable 2.

### Analyses

A latent Dirichlet allocation (LDA) model was developed to identify different subtypes of depression based on patient symptoms. LDA is a topic modeling method; it was chosen in order to reflect the fact that the underlying data was text.

LDA decomposes individual patient symptom data into mixtures of distributions. Here, distributions were seen as subtypes of depression, where each distribution predicts the likelihood of the presence of each symptom. A more detailed introduction to LDA is included in Supplementary eFig. 1.

The number of subtypes, *n*, are not known a priori, and were chosen primarily by comparing model outputs between 2 to 8 subtypes for construct validity within the co-author team. Perplexity, a common metric for evaluating language models, was also used. However, it produced ambiguous results that were not helpful in this context; more details can be found in Supplementary eTable 3. Subtypes were chosen prior to any evaluation of predictive validity.

After the number of subtypes were chosen, k-means clustering was used to create patient groups based on the decomposed data produced by the final LDA model. K-means clustering creates a predetermined number of clusters that minimize variance between data points. The number of clusters was chosen to be *n* to reflect the notion that patients can be described by a single subtype of depression.

The process of producing patient groups is illustrated in Supplementary eFig. 2. Both LDA (sklearn.decomposition.LatentDirichletAllocation) and k-means clustering (sklearn.cluster.KMeans) were performed using version 0.22 of sci-kit learn^22^, a machine learning package for Python 3. Outside of the number of subtypes, the default settings for both classes were used.

After the final model was chosen, demographic and clinical characteristics were then compared between groups using chi-squared tests, evaluating first all derived groups. Afterwards, another subsample of the groups deemed mildest was evaluated to determine whether observed group differences persisted at this level. Presence or absence of events (crisis, emergency presentation, mortality) and mean service use (days active, number of contacts) were similarly compared. Regression analyses were then used to compare outcomes between groups, adjusting all models for age, gender, ethnic group and neighborhood deprivation score: logistic regression (generating odds ratios) for crisis event and emergency presentation, Poisson regression (generating incidence rate ratios) for days active and number of contacts.

## Results

### Subtype selection

A sample of 18,314 patients fulfilled the inclusion criteria. Their symptom data were used to create LDA models, where the final model featured 5 subtypes. Model evaluation was conducted in the Spring of 2020. The final model was chosen mid-June. More information on the patterns observed in the other models are included in Supplementary eTable 4.

**Figure 1:**
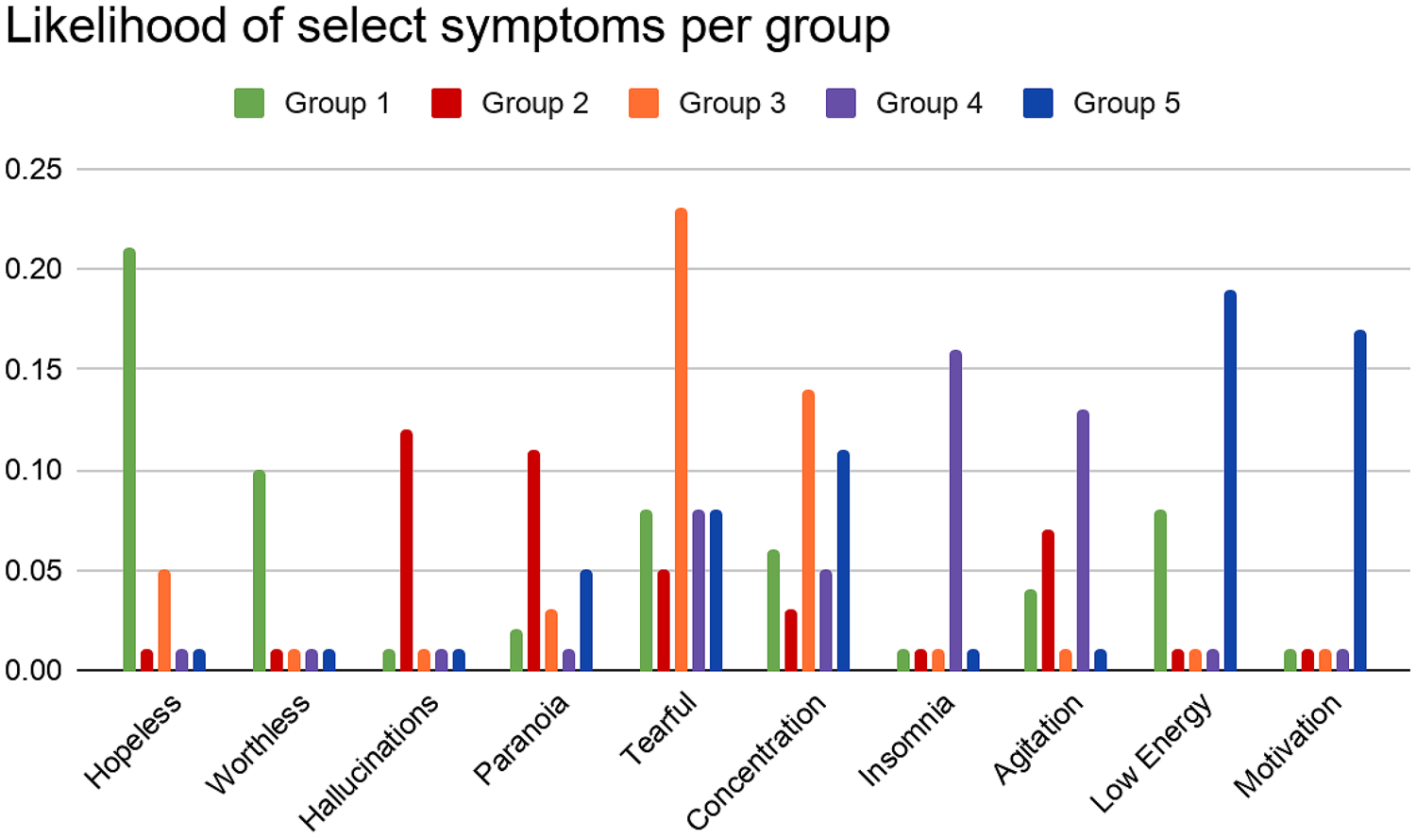
Probabilities of symptoms per patient group. The top two most likely symptoms per group were included.

Each subtype can be characterized by distributions of symptoms. Figure 1 illustrates the differences between distributions by comparing the likelihood of the top two symptoms per subtype. Complete distribution information is included in Supplementary eTable 5.

For the purpose of labelling groups, two presentations were judged to form a severe set. Group 1 had an average of 7.11 (*s* = 3.95) recorded symptoms and Group 2 had 8.62 (*s* = 5.58). On the other hand, Groups 3, 4, and 5 had on average 5.99 (*s* = 3.0), 5.70 (*s* = 4.85), and 4.50 (*s* = 2.79) recorded symptoms respectively. Thus Groups 1 and 2 were viewed as forming a severe set, and Groups 3, 4, and 5 as forming a mild set.

Group 1 was felt to be more reflective of severe emotional distress given its emphasis on hopelessness and worthlessness. On the other hand, Group 2 featured psychotic symptoms, such as hallucinations, more prominently. Thus Group 1 was labelled severe-typical and Group 2 psychotic.

Distinct features were also identified for the milder set. Group 3 was characterized by tearfulness and poor concentration, the most common symptoms in the cohort, as the primary symptoms. Additionally, because hopeless and worthless ideation were unlikely amongst this subtype, it was labelled as mild-typical. Group 4 was labelled an agitated subtype as insomnia, agitation, and aggression were its most common features. Finally, the prominence of low energy and poor motivation in Group 5 supported an anergic-apathetic label.

### Group analysis

Table 1 presents demographic information for each group; Table 2 presents adjusted regression analyses of group outcomes; Table 3 presents HoNOS problems. Comparisons between group outcomes and unadjusted analyses are included in Supplementary eTable 6; analyses for the mild set are presented in Supplementary eTables 7 and 8. Supplementary eTable 9 presents the years in which patients were first active at SLaM. Each table presents *p* values for the total sample as well as the mild set. The differences presented here are significant for both cases unless otherwise noted.

**Table 2:** Strength of the association, adjusted, between various outcomes with each symptom group versus the rest of the sample.

	Severe, typical	Psychotic	Mild, typical	Agitated	Anergic, apathetic
Survival (HR^a^) time to death	**1.24 (1.12,1.37)** < 0.001	1.05 (0.95, 1.16) .34	**0.86 (0.79, 0.95)** < 0.001	0.97 (0.88, 1.06) 0.51	0.96 (0.87, 1.07) 0.46
Crisis event (OR^b^)	1.14 (0.98, 1.33) 0.08	**2.45 (2.15, 2.80)** < 0.001	**0.49 (0.41, 0.57),** < 0.001	0.96 (0.86, 1.13) 0.82	**0.64 (0.54, 0.78))** < 0.001
Emergency presentations (OR)	1.16 (1.05, 1.29), 0.01	**1.29 (1.17, 1.43),** < 0.001	**0.86 (0.78, 0.94)** < 0.001	**0.83 (0.75, 0.92)** < 0.001	1.01 (0.91, 1.13) 0.83
Number of days active in SLAM (IRR^c^)	**0.92 (0.91, 0.93)** < 0.001	**1.14 (1.13, 1.15)** < 0.001	**0.98 (0.97, 0.99)** < 0.001	**0.98 (0.97, 0.99)** < 0.001	1.00 (0.99, 1.01) 0.35
Number of face-to-face contacts (IRR)	1.00 (0.98, 1.02) 0.68	**1.52 (1.49, 1.54)** < 0.001	**0.81 (0.80, 0.82)** < 0.001	**0.87 (0.85, 0.89)** < 0.001	**0.87 (0.85, 0.89),** < 0.001

*Adjusted for age, gender, ethnicity, and index of multiple deprivation score. ^a^Hazard ratio. ^b^Odds ratio. ^c^Incidence rate ratio.

Statistically significant results are bolded. [HR/ OR/ IRR (95% CI), *P*-value].

**Table 3:** Prevalence of HoNOS problems compared by depression sub-group.

Scale	Total 18,314	Group 1, ‘severe, typical’	Group 2, ‘psychotic’	Group 3, ‘mild, typical’	Group 4, ‘agitated’	Group 5, ‘anergic, apathetic”	Total *p*-value^a^	Group 3, 4, and 5 *p*-value^b^
Agitation	1397 (7.6)	180 (5.7)	**442 (14.4)**	282 (5.8)	358 (8.3)	*135 (4.5)*	< 0.001	< 0.001
Self-Injury	2624 (14.3)	**612 (19.5)**	490 (16.0)	561 (11.6)	623 (14.5)	*338 (11.3)*	< 0.001	< 0.001
Drug Misuse	1403 (7.7)	261 (8.3)	**290 (9.5)**	327 (6.8)	329 (7.7)	*196 (6.6)*	.01	.08
Cognition	1,328 (7.3)	193 (6.1)	**364 (11.9)**	*286 (5.9)*	289 (6.7)	196 (6.6)	< 0.001	.43
Physical Illness	3,846 (21.0)	696 (22.2)	**693 (22.7)**	*954 (19.7)*	890 (20.7)	613 (20.6)	.06	.61
Hallucinations	1178 (6.4)	119 (3.8)	**699 (22.9)**	*94 (1.9)*	179 (4.2)	87 (2.9)	< 0.001	< 0.001
Depressed	9,063 (49.5)	1616 (51.5)	**1634 (53.4)**	*2243 (46.3)*	2033 (47.4)	1537 (51.6)	< 0.001	< 0.001
Relationship	3,685 (20.1)	691 (22.0)	**709 (23.2)**	925 (19.1)	822 (19.2)	*538 (18.1)*	< 0.001	.03
Daily Living	3,130 (17.1)	553 (17.6)	**635 (20.8)**	*689 (14.2)*	726 (16.9)	527 (17.7)	< 0.001	< 0.001
Living Conditions	1,714 (9.4)	355 (11.3)	**391 (12.8)**	*363 (7.5)*	347 (8.1)	258 (8.7)	< 0.001	.53
Occupational	3,304 (18)	619 (19.7)	**676 (22.1)**	*728 (15.0)*	750 (17.5)	531 (17.8)	< 0.001	.01
HoNOS Missing	10,704 (58.4)	1798 (57.3)	**2027 (66.3)**	*2680 (55.3)*	244 (57)	1751(58.8)	< 0.001	< 0.001

^a^Chi-squared test with 4 degrees of freedom. ^b^Chi-squared test with 2 degrees of freedom.

Groups with the highest likelihood of problems are presented in bold font and the lowest likelihood in italicized font.

#### Demographic information

Differences in demographic information, as seen in Table 1, were mostly significant across the groups. However, there were no significant differences between the first year that patients were active at SLaM, and no significant differences in mean deprivation score.

There was a gender gap skewing towards women for every group. In the total sample, the difference was 24.3% (62.1% female versus 37.8% male). The largest gender gap was exhibited by the mild-typical group with a difference of 42.1% (71.0% female versus 28.9% male). The smallest gender gap was exhibited by the psychotic group, with a difference of 11.5% (55.7% female versus 44.2% male).

Group differences in ethnicity were statistically significant across the total sample, but not within the mild set. The largest differences were within the psychotic group. White patients were underrepresented; they made up 54.0% of the psychotic group even though they comprised 57.1% of the total sample. Asian patients were overrepresented (6.2% versus 5.0%); Black patients were also overrepresented (18.7% versus 14.9%). Differences in other groups were small, often less than half a percent in magnitude.

With respect to the ages amongst the total sample within groups, the mild-typical and agitated groups featured more patients under the age of 18; patients over the age of 49 were more likely to be a part of the psychotic group; the opposite was true for patients under the age of 18; patients between the ages of 18 and 34 were 3.9% more prominent in the apathetic-anergic group (36.4% versus 32.5%).

#### Group outcomes

Generally, patients within the severe set had worse outcomes than the mild set, as seen in Table 2. For example, patients in the severe-typical group had the highest mortality within the outcomes window (HR = 1.24, 95% CI = 1.12 to 1.37, *p* < 0.001) and mild-typical patients demonstrated the lowest mortality (HR = 0.86, 95% CI = 0.79 to.095, *p* < 0.001). Patients in the psychotic group were the most likely to have a crisis event (OR = 2.45, 95% CI = 2.15 to 2.80, *p* < 0.001), and those within the anergic-apathetic group were less likely to have this outcome (OR = 0.64, 95% CI = 0.54 to 0.77, *p* < 0.001). The same was true for emergency presentations between patients in the psychotic group compared to those in the agitated group.

The severe-typical patients diverged from psychotic patients with respect to the last two outcomes: days active at SLaM and number of face-to-face contacts. They were closer to the mild set, which tended to have fewer active days at SLaM; the severe-typical group had the fewest active days. On the other hand, the psychotic group engaged with SLaM the most. They had the most days active in SLaM (IRR = 1.14, 95% CI = 1.13 to 1.15, *p* < 0.001) and the most face-to-face contacts (IRR = 1.52 95% CI = 1.54 to 1.15, *p* < 0.001).

#### HoNOS problems

HoNOS problems were well-aligned with the primary symptoms of each subtype. For example, patients in the psychotic group had the most HoNOS problems, with the exception of self-injury, which was more common in the severe-typical group. And compared to every other group, patients in the mild set generally displayed fewer HoNOS problems. However, drug misuse and physical illness were not significantly different. Differences in several HoNOS problems were insignificant within the mild set: drug misuse, cognition, physical illness, depression, living conditions, and occupation. The primary differences within the mild set was the higher prevalence of some symptoms amongst the agitated group relative to the mild-typical group and the lower prevalence in the anergic-apathetic group.

## Discussion

### Construct validity

In this study, we identified depressive subtypes in symptom data derived from unstructured EHRs. Five distinct subtypes were identified based upon patient data collected within a month after an initial face-to-face encounter with SLaM: severe-typical, psychotic, mild-typical, agitated, and anergic-apathetic. They were then used to create patient groups for validation. To this end, follow-up characteristics and outcomes recorded at least 3 months after the initial window were studied. Outcomes were extracted and evaluated after the subtypes had been created and finalized.

Each subtype was defined by several symptoms that were not prominent in any other group and were well-characterized from a qualitative perspective. In other words, subtypes were more representative of the way clinicians described their patients. Moreover, they were predictive of a variety of future outcomes, such as crisis events, emergency presentations, likelihood to be deceased, as well as service utilization. Unsurprisingly, this was especially true for the psychotic and mild-typical groups.

Subtypes were aligned well with future mental and behavioral issues found in the structured data: patients in the severe-typical group had more problems with self-injury; those in the psychotic group had more hallucinations problems rated on the HoNOS structured instrument; patients in the mild-typical group had the fewest problems. Compared within the mild set, patients in the anergic-apathetic group were more likely to be described as depressed; agitated patients were more likely to have HoNOS problems.

These results are reflective of some patterns found in the clinical literature. For example, several studies have found that African American patients are more likely to be described as exhibiting hallucinatory behavior and seek treatment for depression at lower rates than Caucasian patients^23–26^. Depression severity is correlated with increased emergency department visits and healthcare utilization^27–29^. Patients most likely to be later described as depressed featured anergia, the second most common residual symptom of depression, and one that poses significant problems for daily living^30^. There was a sizable gender gap favoring women in every group, but this gap was the smallest amongst the psychotic group. This finding aligns with existing research that suggests that unlike mood or anxiety disorders, the prevalence of psychosis is approximately even between men and women^31,32^.

However, our findings showed some inconsistencies with other studies. For example, there were no statistically significant differences in problems with physical illness between groups, even though associations with physical illness and depression severity have been reported^33^. Intuitively, problems with daily living and living conditions might have been expected to differ between groups, yet significant differences only existed for the former within the mild set. The number of patients per group was spread reasonably evenly, though the rate of different types of depression need not be distributed in this way^33–38^. Severe-typical patients were not that likely to have an emergency presentation, considering the number of outcome variables, even though severity is correlated with hospitalization^27,39^. Similarly, severity was not as predictive of drug misuse problems on the HoNOS scale compared to other outcomes, though this has been reported for substance abuse broadly^40^.

Additionally, some factors do not lend themselves to easy interpretation. For example, a significantly large gender gap was present in the mild-typical group relative to those in the other subtypes within the mild set; the causes of this gap can be attributed to multiple reasons, but exactly which combination is not possible to discern. And while the results presented here are statistically significant, some are smaller in magnitude than what may be expected, such as the odds of having an emergency presentation: severe, typical patients were only 1.17 times more likely than their mild counterparts; however, this might reflect the fact that all patients were receiving care from a specialist mental health service, so represent a relatively severe subset of all community cases of depression, potentially diluting differences between symptom cluster groups. There are also issues of representation, such as the differences in the availability of HoNOS scores.

As a result of these discrepancies, it is both true that these subtypes provide clinically relevant information, but they should be still understood as complementary to current diagnostic tools.

### Study context

Previous studies have focused on studying small samples of patients with a narrow set of depressive symptoms. They typically employ latent class analyses and factor analysis to identify subtypes, though some also use k-means clustering^5,6,41–44^. Generally, groups are stratified across severity. For example, one LCA study^45^ produced the following groups: “severe typical”, “mild typical”, “severe atypical”, “mild atypical”, “intermediate”, and “minimal symptoms”. A k-means study identified a “vital” and “nonvital” group amongst depressed men, where individuals in the former were more likely to have each symptom compared to those in the latter. We address these issues, in part, by analyzing a large cohort and including a broader set of symptoms.

This study also differed in that the underlying data comprised free text recorded by clinicians, as opposed to checklists from research instruments applied to screened samples. While clinical text has been analyzed in other medical specialties, it has seen limited use for depression, though text mining for psychiatry has seen increased use within the last decade^46^. It is not clear, a priori, what types of information are important for different applications. Moreover, clinicians often write narratives about their patients, as opposed to any set of semi-structured information, such as a list of symptoms or surgeries. As a result, contextual issues make accurate data extraction difficult^47^; research to this end is also hampered due to a lack of data access within healthcare settings^48^.

Here, we have shown that the symptom data captured by clinicians can be used to define meaningful constructs to categorize patient experiences in early stages of specialist care. In particular, the constructs are qualitative in nature—they relate directly to patient symptoms—and are relevant to future outcomes. Thus, unstructured EHRs for this task merit further exploration.

One approach could involve studying how to better identify constructs. In this study, one set of subtypes was chosen for further analysis based upon potential clinical use, i.e. the subtypes should describe clinically relevant patient profiles, and not goodness-of-fit, which poses issues surrounding model interpretability. K-means clustering was used to group patients, but other methods, like organizing patients based upon their most prominent subtype, could have been used. Realistically, many patients will not fit cleanly into one subtype; allowing for additional clusters could let patient groups with more complicated profiles to emerge.

Subtypes should also be leveraged to predict a broader range of outcomes, such as medication efficacy. One way to do so is to simply extract a wider range of symptoms as well as other relevant characteristics in unstructured EHRs. This can also include information commonly collected from depression scales, such as symptom temporality or severity. To the latter point, prior analyses with structured data have already created promising predictive models for treatment response^49,50^.

### Limitations

This study has several limitations. First, the choice of symptoms was limited in scope. While new variables are constantly being extracted from CRIS, some symptoms classically associated with depression, such as anxiety, were not available for use in this study. This biases which subtypes can be derived from the data. For example, mood reactivity and weight gain are two symptoms that have not been extracted, making it difficult to identify and study atypical depression in this cohort.

Second, like other cluster analyses, the results presented here are sensitive to methodological changes. For example, if ten groups were chosen over five, the differences between groups may have been too slight to detect. Alternatively, fewer groups could have been generated, potentially obscuring important subtypes. Had we chosen two groups, distinctions between depressed patients with moderate, severe, or psychotic symptoms would be harder to detect.

Third, patients treated in a setting like SLaM will have more severe mental health issues, since all will have either been first seen and referred by a general practitioner or will have been identified as emergency care presentations. The results presented here are specific to patients diagnosed primarily with and treated for their depression. This excludes several relevant populations, including patients with a different primary diagnosis and people that have depression yet have not sought yet treatment.

Additionally, noise is introduced into unstructured EHRs from several different sources. The symptom data here is less precise than information provided by depression scales, which track the severity of individual items, whereas entities extracted from clinical text tend to be binary: present or not recorded. Scales also specify time periods, e.g. within the previous 2 weeks, whereas it is generally difficult to extract temporal relations from text. Moreover, clinicians do not record information consistently. For example, questionnaires will always include an item for low mood or lack of interest, but this information was not always recorded for patients in this study.

## Conclusion

In this study, we decomposed depression, a highly heterogeneous disorder, into 5 subtypes using a broad set of symptom data derived from unstructured EHRs. Previous studies have typically relied on a limited set of symptoms related to depression, whereas symptoms used here included those related to psychosis and bipolar in addition to depression. These subtypes—severe-typical, psychotic, mild-typical, agitated, and anergic-apathetic—were created using an unsupervised latent model and validated by examining their relationship to a variety of different clinical outcomes, including those that captured future health conditions. Broadly, these subtypes tended to be significantly different in ways that corresponded well to their defining symptom. For example, subtypes that were intuitively severe tended to have more mental and behavioral problems compared to milder presentations. Thus, they were clinically relevant, and given that they were automatically generated, could potentially be implemented in different settings to guide clinicians. Additionally, by focusing on data in unstructured EHRs, which include symptoms not captured by depressive scales, opens new avenues to study depression in relation to other disorders. To these ends, future work could focus on more clinical outcomes, such as antidepressant efficacy, and leveraging more information, such as more symptom data, different data sources, or a more holistic use of clinical text.

## Supplementary Information

Supplementary Information.

## Data Availability

Data from this study is not publicly available, but access can be obtained by contacting the Clinical Record Interactive Search (CRIS) team.
