# Supplementary material for: Identifying subtypes of depression in clinician-annotated text: a retrospective cohort study
Source: Sci Rep. 2021 Nov 17;11:22426. doi: 10.1038/s41598-021-01954-4 (PMC8599474; doi:10.1038/s41598-021-01954-4)
Supplement: Supplementary file 1 — Supplementary Information. [file 41598_2021_1954_MOESM1_ESM.docx]

**Supplementary Materials**

**Identifying subtypes of depression in clinician-annotated text:**

**A retrospective cohort study**

| Benson Kung^1^*  benson@prairiehealth.co | Maurice Chiang^1^  maurice@prairiehealth.co |
| --- | --- |

| Gayan Perera^2^  gayan.perera@kcl.ac.uk | Megan Pritchard^2,3^  megan.pritchard@kcl.ac.uk | Robert Stewart^2,3^  robert.stewart@kcl.ac.uk |
| --- | --- | --- |

1. Prairie Health, Palo Alto, United States of America

2. King’s College London, Institute of Psychiatry, Psychology and Neuroscience, London, United Kingdom

3. South London and Maudsley NHS Foundation Trust, London, United Kingdom

**eTable 1:** Psychiatric symptoms used for subtype identification

| Anergia | Anhedonia | Apathy | Aggression | Agitation |
| --- | --- | --- | --- | --- |
| Arousal | Blunted Affect | Circumstantial Speech | Concrete Thinking | Delusions |
| Derailment of Speech | Disturbed Sleep | Echolalia | Elation | Flight of Ideas |
| Formal Thought Disorder | Grandiosity | Guilt | Hallucinations | Helplessness |
| Hopelessness | Hostility | Insomnia | Irritability | Loss of Coherence |
| Low Energy | Low Mood | Mood Instability | Mutism | Negative Symptoms |
| Paranoia | Passivity | Persecutory Ideation | Poor Appetite | Poor Concentration |
| Poor Insight | Poor Motivation | Poverty of Speech | Poverty of Thought | Social Withdrawal |
| Stupor | Suicidal Ideation | Tangential Speech | Tearfulness | Thought Block |
| Thought Broadcast | Thought Insertion | Thought Withdrawal | Treatment Resistant Depression | Waxy Flexibility |
| Weight Loss | Worthlessness |  |  |  |

Almost every symptom extracted from the Clinical Record Interactive Search (CRIS), as of September 2020, was used to create models.

Two symptoms from the above list were not used — “Low Mood” and “Disturbed Sleep” — because they were featured by every subtype in our initial analyses and were not informative. This was unsurprising given that they were more prevalent than other symptoms: “Low Mood” was used to describe 85% of patients and “Disturbed Sleep” was used to describe 61%.

Note that some relevant symptoms, such as weight gain, have not been extracted from CRIS, and therefore were not available for use in this study.

Typically, with topic modeling methods, words are filtered prior to model creation. However, this is not necessarily better than removing words after model creation (Schofield et al. 2017).

Schofield, Alexandra, Måns Magnusson, and David Mimno. "Pulling out the stops: Rethinking stopword removal for topic models." *Proceedings of the 15th Conference of the European Chapter of the Association for Computational Linguistics: Volume 2, Short Papers*. 2017.



**eTable 2:** Medications received

|  | Full sample | Groups 2-4 | 0 | 1 | 2 | 3 | 4 | *p*-value  Full Sample | *p*-value  Groups 2-4 |
| --- | --- | --- | --- | --- | --- | --- | --- | --- | --- |
| **Total Sample** | 18,314 | 12,115 | 3,140 | 3,059 | 4,844 | 4,291 | 2,980 |  |  |
| **Medications received** |  |  |  |  |  |  |  |  |  |
| Antipsychotics | 1849 (10.1) | 821 (6.8) | 238 (7.6) | 790 (25.8) | 225 (4.6) | 400 (9.3) | 196 (6.6) | <0.001 | <0.001 |
| Mood stabilizers | 505 (2.8) | 287 (2.4) | 98 (3.1) | 120 (3.9) | 101 (2.1) | 101 (2.4) | 85 (2.9) | <0.001 | 0.10 |
| Antidepressants | 5870 (32.1) | 3665 (30.3) | 990 (31.5) | 1215 (39.7) | 1346 (27.8) | 1362 (31.7) | 957 (32.1) | <0.001 | <0.001 |
| Hypnotics and anxiolytics | 1737 (9.5) | 992 (8.2) | 293 (9.3) | 452 (14.8) | 320 (6.6) | 443 (10.3) | 229 (7.7) | <0.001 | <0.001 |
| **Number of types of antidepressants received** |  |  |  |  |  |  |  | <0.001 | <0.001 |
| 1 | 4120 (22.5) | 2595 (21.4) | 677 (21.6) | 848 (27.7) | 984 (20.3) | 956 (22.3) | 655 (22) |  |  |
| 2 | 1300 (7.1) | 789 (6.5) | 218 (6.9) | 293 (9.6) | 272 (5.6) | 304 (7.1) | 213 (7.1) |  |  |
| 3 | 312 (1.7) | 202 (1.7) | 61 (1.9) | 49 (1.6) | 63 (1.3) | 69 (1.6) | 70 (2.3) |  |  |
| 4 or more | 138 (0.8) | 79 (0.7) | 34 (1.1) | 25 (0.8) | 27 (0.6) | 33 (0.8) | 19 (0.6) |  |  |

**eFigure 1:** Brief introduction to Latent Dirichlet Allocation


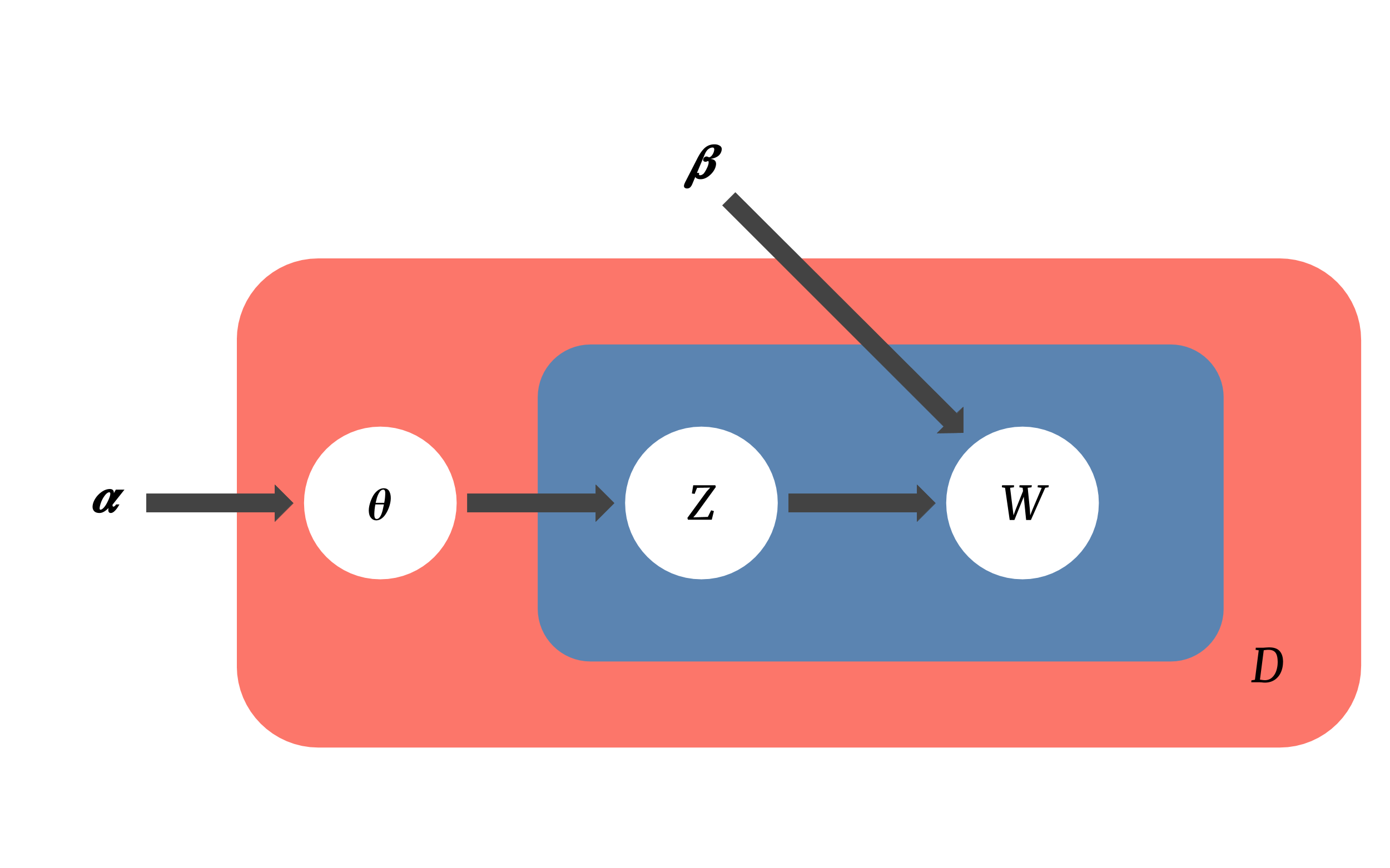


Latent Dirichlet Allocation (LDA) was developed by Blei et al.^a^ to model “collections of discrete data”. It is a “three-level hierarchical Bayesian model”. The classic use-case of LDA is to model text corpora, which will be used here as an illustrative example. In the case of a collection of documents, LDA views each document as a mixture of topics: “what does each document discuss?”. Each topic is then seen as a distribution of words: “which words are used to discuss a particular topic?”

For example, consider the politics section of a newspaper. As a whole, the articles will discuss a variety of topics, such as elections. But individual articles often cover more than one topic. In the case of elections, it is likely that political parties and lawsuits will also be discussed. Thus, one article can be understood as a combination of the topics “elections”, “lawsuits”, and “political parties”. But certain words, such as “supreme court”, will be used more often in “lawsuits”, though they will also be used in the other two topics^b^. To reflect this, LDA views topics as distributions of words, where each word has some probability to be used. As a result, it allows for an extra level of granularity: “supreme court” will have a higher probability of being used in “lawsuits”, but a smaller probability in “elections” and “political parties”.

More formally, as seen in the figure, LDA assumes that documents are created in the following fashion:

1. Use the Dirichlet prior $\boldsymbol{\alpha}$ to create a document ***D***, i.e. a mixture of topics $\boldsymbol{\theta}$**.**
2. Choose a word by
   1. Picking a topic ***Z*** from $\boldsymbol{\theta}$ and
   2. Picking a word ***W*** from a distribution based upon the topic ***Z*** and the Dirichlet prior $\boldsymbol{\beta}$.

In this study, the symptom data is analogous to the collection of documents; patients to documents; depressive subtypes as topics.

^a^Blei, David M., Andrew Y. Ng, and Michael I. Jordan. "Latent dirichlet allocation." *Journal of machine Learning research* 3. Jan (2003): 993-1022

^b^For non-Americans, the Supreme Court is the highest court in the country and is intricately tied to America’s two-party system. For example, political parties play a major role in choosing who is on the Supreme Court. Elections are naturally tied to political parties.

**eTable 3:** Perplexity scores

| ***n* topics** | **Perplexity** |
| --- | --- |
| 2 | 28.1 |
| 3 | 30.4 |
| 4 | 32.5 |
| 5 | 34.4 |
| 6 | 36.0 |
| 7 | 37.4 |
| 8 | 38.5 |

Perplexity is a metric commonly used to evaluate topic models like latent Dirichlet allocation. Generally speaking, lower values indicate better model fit; in this case, we might choose the model with two topics because it has the lowest perplexity. However, in terms of clinical utility, two topics were deemed insufficient; the two-topic model only differentiated between depression and depression with psychotic features. As a result, perplexity was not used to choose the final model for this study. See **eTable 4** for more information on the other models.

**eFigure 2:** Study overview

**
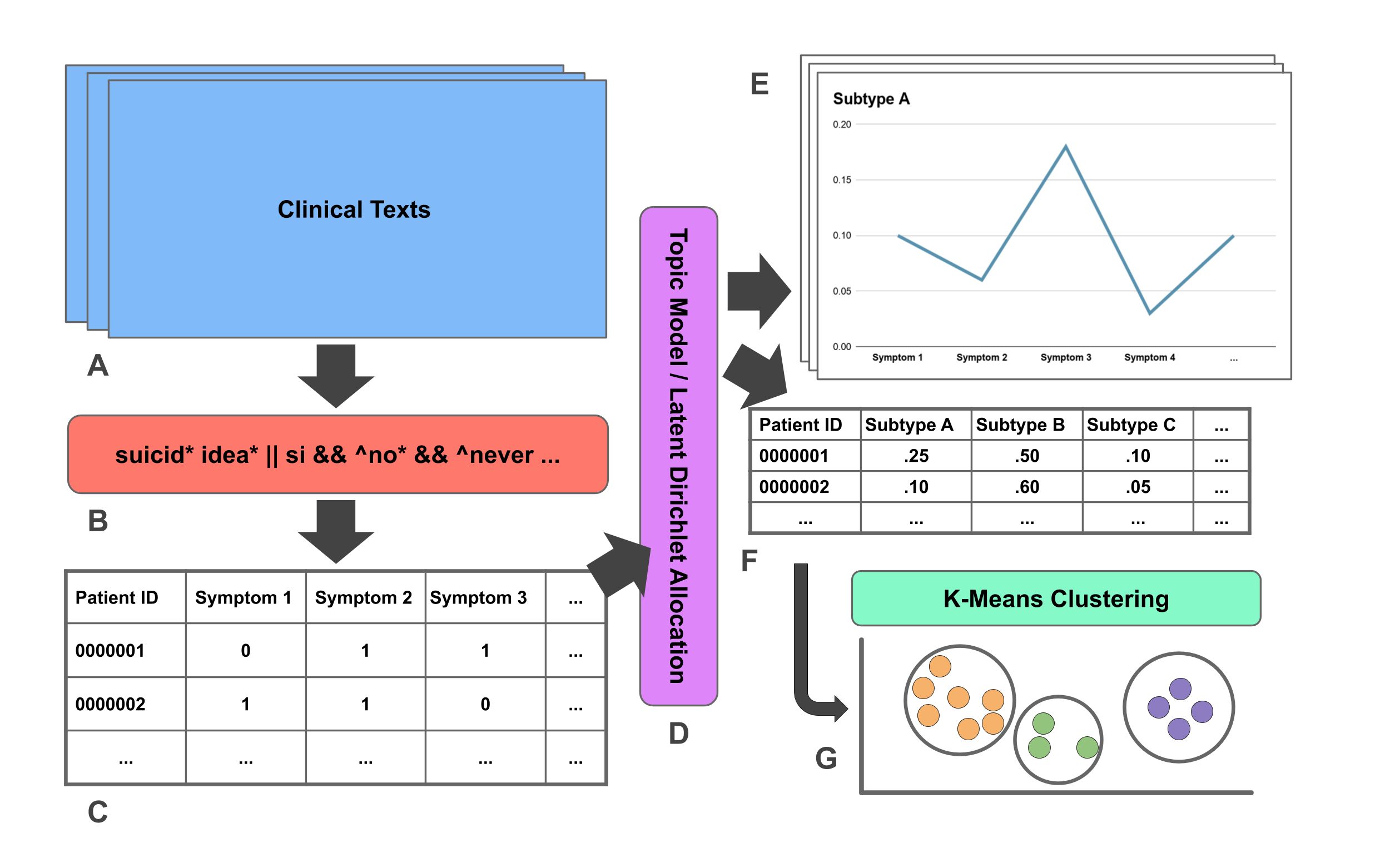
**

(A) Clinical texts from patients diagnosed with depression (ICD-10 F32 or F33) were obtained. (B) Rules-based algorithms, created prior to this study, were used to identify properly contextualized mentions of various symptoms. (C) Patients were then represented with binary variables, where a symptom was considered present if mentioned by a clinician within a month of a patient’s initial face-to-face contact with SLaM. (D) Symptom data was passed through a topic model which produced (E) symptom distributions for each subtype and (F) patients represented by mixtures of subtypes. Reduced patient data was then grouped with (G) k-means clustering for outcome analysis.

**eTable 4:** Symptom probabilities for models featuring 2 to 8 subtypes

| n | Primary Symptoms and Probabilities | | | | |
| --- | --- | --- | --- | --- | --- |
|  |  |  |  |  |  |
| 2 | Tearful  .11 | Paranoia  .10 | Mood Instability  .09 | Agitation  .08 | Hallucination  .07 |
|  | Hopelessness  .11 | Suicidal Ideation  .10 | Poor Concentration  .10 | Low Energy  .09 | Tearful  .09 |
| 3 | Paranoia  .10 | Hallucination  .09 | Agitation  .09 | Tearful  .08 | Aggression  .07 |
|  | Hopelessness  .19 | Suicidal Ideation  .12 | Tearful  .11 | Guilt  .09 | Worthlessness  .08 |
|  | Poor Concentration  .12 | Low Energy  .11 | Tearful  .10 | Poor Motivation  .10 | Mood Instability  .10 |
| 4 | Paranoia  .12 | Hallucination  .11 | Agitation  .08 | Poor Insight  .08 | Delusion  .07 |
|  | Hopelessness  .23 | Suicidal Ideation  .10 | Worthlessness  .10 | Tearful  .09 | Helplessness  .08 |
|  | Low Energy  .18 | Poor Motivation  .18 | Poor Concentration  .11 | Anhedonia  .10 | Mood Instability  .08 |
|  | Tearful  .22 | Poor Concentration  .11 | Guilt  .09 | Irritability  .08 | Mood Instability  .08 |
| 5 | Hallucination  .12 | Paranoia  .11 | Delusion  .08 | Agitation  .07 | Poor Insight  .07 |
|  | Hopelessness  .21 | Worthlessness  .10 | Suicidal Ideation  .10 | Helplessness  .09 | Tearful  .08 |
|  | Low Energy  .19 | Poor Motivation  .17 | Poor Concentration  .11 | Anhedonia  .09 | Tearful  .08 |
|  | Insomnia  .16 | Agitation  .13 | Aggression  .11 | Tearful  .08 | Mood Instability  .06 |
|  | Tearful  .23 | Poor Concentration  .14 | Guilt  .13 | Irritability  .10 | Mood Instability  .10 |
| 6 | Hallucination  .12 | Paranoia  .11 | Delusion  .08 | Poor Insight  .07 | Agitation  .07 |
|  | Hopelessness  .19 | Worthlessness  .14 | Helplessness  .11 | Tearful  .09 | Low Energy  .08 |
|  | Poor Motivation  .21 | Low Energy  .12 | Mood Instability  .12 | Poor Concentration  .11 | Tearful  .10 |
|  | Aggression  .18 | Insomnia  .13 | Agitation  .11 | Tearful  .07 | Mood Instability  .07 |
|  | Tearful  .22 | Guilt  .14 | Poor Concentration  .14 | Irritability  .11 | Weight Loss  .10 |
|  | Anhedonia  .19 | Blunted Affect  .12 | Suicidal Ideation  .11 | Poor Concentration  .08 | Hopelessness  .08 |
| 7 | Paranoia  .10 | Hallucination  .10 | Poor Insight  .09 | Delusion  .08 | Agitation  .07 |
|  | Hopelessness  .16 | Worthlessness  .15 | Helplessness  .13 | Tearful  .07 | Low Energy  .07 |
|  | Poor Motivation  .24 | Low Energy  .18 | Poor Concentration  .13 | Mood Instability  .11 | Tearfulness  .09 |
|  | Aggression  .20 | Insomnia  .14 | Agitation  .09 | Tearfulness  .08 | Mood Instability  .07 |
|  | Poor Concentration  .17 | Weight Loss  .15 | Irritability  .14 | Tearful  .14 | Guilt  .07 |
|  | Anhedonia  .21 | Blunted Affect  .12 | Suicidal Ideation  .11 | Poor Concentration  .08 | Hopelessness  .08 |
|  | Tearful  .28 | Guilt  .16 | Paranoia  .12 | Hopelessness  .10 | Suicidal Ideation  .10 |
| 8 | Agitation  .15 | Poor Insight  .14 | Tearful  .08 | Mood Instability  .06 | Paranoia  .06 |
|  | Worthlessness  .17 | Hopelessness  .17 | Helplessness  .14 | Low Energy  .07 | Tearful  .07 |
|  | Poor Motivation  .26 | Low Energy  .17 | Poor Concentration  .12 | Mood Instability  .11 | Tearful  .08 |
|  | Aggression  .17 | Insomnia  .17 | Agitation  .08 | Tearful  .07 | Mood Instability  .06 |
|  | Irritability  .21 | Weight Loss  .16 | Poor Concentration  .15 | Tearful  .12 | Suicidal Ideation  .06 |
|  | Anhedonia  .20 | Blunted Affect  .11 | Suicidal Ideation  .10 | Poor Concentration  .08 | Hopelessness  .08 |
|  | Tearful  .29 | Guilt  .19 | Suicidal Ideation  .11 | Hopelessness  .11 | Paranoia  .08 |
|  | Hallucination  .17 | Paranoia  .14 | Delusion  .13 | Persecution  .08 | Agitation  .06 |

Each row is a different subtype. The first five symptoms ordered by likelihood are presented for each subtype.

LDA models with 2 to 4 subtypes were deemed to be insufficient; they lacked the mild-typical and agitated subtypes included in other subtypes. LDA models with 5 to 8 models were deemed unnecessary; they produced subtypes too closely related.

**eTable 5:** Final model symptom distribution

| Topic | Symptom Probabilities | | | | |
| --- | --- | --- | --- | --- | --- |
| 1 | Hopelessness  .22 | Worthlessness  .10 | Suicidal Ideation  .10 | Helplessness  .09 | Tearful  .08 |
|  | Low Energy  .08 | Guilt  .07 | Poor Concentration  .06 | Anhedonia  .05 | Agitation  .04 |
| 2 | Hallucination  .12 | Paranoia  .11 | Delusion  .08 | Agitation  .07 | Poor Insight  .07 |
|  | Persecution  .05 | Tearful  .05 | Aggression  .05 | Suicidal Ideation  .04 | Poor Concentration  .03 |
| 3 | Tearful  .23 | Poor Concentration  .14 | Guilt  .13 | Irritability  .10 | Weight Loss  .10 |
|  | Suicidal Ideation  .09 | Mood Instability  .08 | Hopelessness  .05 | Aggression  .03 | Paranoia  .03 |
| 4 | Insomnia  .16 | Agitation  .13 | Aggression  .11 | Tearful  .08 | Mood Instability  .06 |
|  | Suicidal Ideation  .05 | Poor Concentration  .05 | Irritability  .05 | Blunted Affect  .04 | Weight Loss  .04 |
| 5 | Low Energy  .20 | Poor Motivation  .17 | Poor Concentration  .11 | Anhedonia  .09 | Tearful  .08 |
|  | Mood Instability  .08 | Suicidal Ideation  .05 | Weight Loss  .05 | Paranoia  .05 | Social Withdrawal  .04 |

Note the top 10 symptoms per topic are presented. The likelihoods are normalized, i.e. if the 50 symptoms were presented, the sum of the likelihoods would be 1. Thus, the average symptom would have a likelihood of .05.

**eTable 6:** Strength of the association, unadjusted, between various outcomes with each symptom group versus the rest of the sample. Statistically significant results are bolded.

|  | Severe, Typical | Psychotic | Mild, Typical | Agitated | Anergic, Apathetic |
| --- | --- | --- | --- | --- | --- |
| Survival (HR^a^) time to death | 1.09 (0.99,1.20), 0.09 | **1.21 (1.10, 1.33), <0.001** | **0.78 (0.71, 0.85), <0.001** | 0.99 (0.91, 1.08), 0.84 | 1.07 (0.97, 1.18), 0.18 |
| Crisis event (OR^b^) | 1.15 (1.00, 1.33), 0.06 | **2.45 (2.16, 2.78), <0.001** | **0.49 (0.42, 0.57), <0.001** | 0.96 (0.84, 1.10), 0.56 | **0.64 (0.54, 0.77), <0.001** |
| Emergency presentations (OR) | 1.12 (1.02, 1.24), 0.02 | **1.26 (1.15, 1.39), <0.001** | 0.88 (0.81, 0.96), 0.01 | **0.85 (0.78, 0.94), <0.001** | 1.00 (0.90, 1.11), 0.98 |
| Number of days active in SLAM (IRR^c^) | **0.90 (0.89, 0.91), <0.001** | **1.13 (1.12, 1.14), <0.001** | 1.01 (1.00, 1.02), 0.01 | **0.97 (0.96, 0.98), <0.001** | 1.00 (0.99, 1.01), 0.97 |
| Number of face-to-face contacts (IRR) | 0.98 (0.96, 1.00), 0.03 | **1.48 (1.46, 1.51), <0.001** | **0.84 (0.82, 0.85), <0.001** | **0.96 (0.95, 0.98), <0.001** | **0.87 (0.86, 0.89), <0.001** |

^a^Hazard ratio. ^b^Odds ratio. ^c^Incidence rate ratio.

**eTable 7:** Strength of the association, unadjusted, between various outcomes with each symptom group versus the mild set. Statistically significant results are bolded.

|  | Mild, Typical | Agitated | Anergic, Apathetic |
| --- | --- | --- | --- |
| Survival (HR^s^) time to death | **0.81 (0.74, 0.90)**  **<0.001** | 1.09 (0.98, 1.20)  0.10 | **1.17 (1.05, 1.30)**  **<0.001** |
| Crisis event (OR^b^) | **0.67 (0.56, 0.80)**  **<0.001** | **1.58 (1.35, 1.86)**  **<0.001** | 0.90 (0.75, 1.09)  0.30 |
| Emergency presentations (OR) | 0.98 (0.89, 1.08)  0.68 | 0.94 (0.85, 1.03)  0.21 | 1.11 (1.00, 1.24)  0.05 |
| Number of days active in SLAM (IRR^c^) | **1.02 (1.01, 10.03**  **<0.001** | **0.98 (0.97, 0.99)**  **<0.001** | **1.01 (1.00, 1.02)**  **<0.001** |
| Number of face-to-face contacts (IRR) | **0.94 (0.92, 0.95)**  **<0.001** | **1.10 (1.08, 1.11)**  **<0.001** | 0.97 (0.96, 0.99)  0.01 |

^a^Hazard ratio. ^b^Odds ratio. ^c^Incidence rate ratio.

**eTable 8:** Strength of the association, adjusted, between various outcomes with each symptom group versus the mild set. Statistically significant results are bolded.

|  | Mild, Typical | Agitated | Anergic, Apathetic |
| --- | --- | --- | --- |
| Survival (HR^a^) time to death | 0.93 (0.84, 1.02)  0.14 | 1.05 (0.95, 1.16)  0.36 | 1.04 (0.93, 1.16)  0.52 |
| Crisis event (OR^b^) | **0.65 (0.55, 0.79)**  **<0.001** | **1.61 (1.36, 1.91)**  **<0.001** | 0.91 (0.74, 1.10)  0.30 |
| Emergency presentations (OR) | 0.97 (0.87, 1.07)  0.51 | 0.93 (0.83, 1.02)  0.15 | 1.15 (1.03, 1.29)  0.02 |
| Number of days active in SLAM (IRR^c^) | 0.99 (0.98, 1.00)  0.04 | 0.99 (0.98, 1.00)  0.01 | **1.02 (1.01, 1.03)**  **<0.001** |
| Number of face-to-face contacts (IRR) | **0.91 (0.89, 0.92)**  **<0.001** | **1.12 (1.10, 1.15)**  **<0.001** | 0.98 (0.96, 1.01)  0.08 |

Adjusted for age, gender, ethnicity, and index of multiple deprivation score. ^a^Hazard ratio. ^b^Odds ratio. ^c^Incidence rate ratio.

**eTable 9:** The years in which patients were first active at SLaM. The *p*-value for the full sample was 0.13, and the *p*-value for Groups 3-5 was 0.03.

|  | Full sample | Groups 3-5 | 1 | 2 | 3 | 4 | 5 |
| --- | --- | --- | --- | --- | --- | --- | --- |
| **Total Sample** | 18,314 | 12,115 | 3,140 | 3,059 | 4,844 | 4,291 | 2,980 |
| **Year of first SLaM contact** | *p* = 0.13 | *p* = 0.03 |  |  |  |  |  |
| 2007 | 1442 (7.9) | 1025 (8.5) | 211 (6.7) | 206 (6.7) | 426 (8.8) | 328 (7.6) | 271 (9.1) |
| 2008 | 1351 (7.4) | 906 (7.5) | 217 (6.9) | 228 (7.5) | 398 (8.2) | 289 (6.7) | 219 (7.3) |
| 2009 | 1384 (7.6) | 942 (7.8) | 221 (7.0) | 221 (7.2) | 406 (8.4) | 293 (6.8) | 243 (8.2) |
| 2010 | 1765 (9.6) | 1195 (9.9) | 278 (8.9) | 292 (9.5) | 501 (10.3) | 408 (9.5) | 286 (9.6) |
| 2011 | 1696 (9.3) | 1127 (9.3) | 283 (9.0) | 286 (9.3) | 435 (9.0) | 428 (10) | 264 (8.9) |
| 2012 | 1691 (9.2) | 1131 (9.3) | 276 (8.8) | 284 (9.3) | 475 (9.8) | 407 (9.5) | 249 (8.4) |
| 2013 | 1685 (9.2) | 1147 (9.5) | 280 (8.9) | 258 (8.4) | 439 (9.1) | 425 (9.9) | 283 (9.5) |
| 2014 | 1418 (7.7) | 944 (7.8) | 252 (8.0) | 222 (7.3) | 355 (7.3) | 353 (8.2) | 236 (7.9) |
| 2015 | 1534 (8.4) | 976 (8.1) | 288 (9.2) | 270 (8.8) | 392 (8.1) | 375 (8.7) | 209 (7.0) |
| 2016 | 1502 (8.2) | 950 (7.8) | 272 (8.7) | 280 (9.2) | 355 (7.3) | 366 (8.5) | 229 (7.7) |
| 2017 | 1513 (8.3) | 911 (7.5) | 306 (9.7) | 296 (9.7) | 344 (7.1) | 318 (7.4) | 249 (8.4) |
| 2018 | 1333 (7.3) | 861 (7.1) | 256 (8.2) | 216 (7.1) | 318 (6.6) | 301 (7) | 242 (8.1) |
